# The anaemia of *Plasmodium vivax* malaria

**DOI:** 10.1186/1475-2875-11-135

**Published:** 2012-04-27

**Authors:** Nicholas M Douglas, Nicholas M Anstey, Pierre A Buffet, Jeanne R Poespoprodjo, Tsin W Yeo, Nicholas J White, Ric N Price

**Affiliations:** 1 Global Health Division, Menzies School of Health Research, PO Box 41096, Casuarina, NT 0811, Australia; 2 Centre for Tropical Medicine, Nuffield Department of Clinical Medicine, University of Oxford, Oxford, UK; 3 Division of Medicine, Royal Darwin Hospital, Darwin, NT, Australia; 4 INSERM - UPMC, (Paris 6 University) UMRs945, F-75013, Paris, France; 5 Department of Parasitology, Pitié-Salpétrière Hospital, Assistance Publique – Hôpitaux de Paris, F-75013, Paris, France; 6 Institut Pasteur, Unité d’Immunologie Moléculaire des Parasites, Département de Parasitologie Mycologie, F-75015, Paris, France; 7 Mimika District Health Authority, Timika, Papua, Indonesia; 8 Papuan Health and Community Development Foundation, Timika, Papua, Indonesia; 9 Faculty of Tropical Medicine, Mahidol University, Bangkok, Thailand

**Keywords:** *Plasmodium vivax*, Malaria, Anaemia, Epidemiology, Pathogenesis

## Abstract

*Plasmodium vivax* threatens nearly half the world’s population and is a significant impediment to achievement of the millennium development goals. It is an important, but incompletely understood, cause of anaemia. This review synthesizes current evidence on the epidemiology, pathogenesis, treatment and consequences of vivax-associated anaemia. Young children are at high risk of clinically significant and potentially severe vivax-associated anaemia, particularly in countries where transmission is intense and relapses are frequent. Despite reaching lower densities than *Plasmodium falciparum*, *Plasmodium vivax* causes similar absolute reduction in red blood cell mass because it results in proportionately greater removal of uninfected red blood cells. Severe vivax anaemia is associated with substantial indirect mortality and morbidity through impaired resilience to co-morbidities, obstetric complications and requirement for blood transfusion. Anaemia can be averted by early and effective anti-malarial treatment.

## Background

*Plasmodium vivax* threatens approximately 2.8 billion people globally and, because of its particular biological characteristics, will be more difficult to eradicate than *Plasmodium falciparum*[1-9]. Over recent years, case series [10-13], surveillance studies [14-17] and reviews [4,18-24] have linked vivax malaria with a number of severe manifestations similar to those found in *P. falciparum* infection; observations that challenge the notion that vivax malaria is a benign disease. The causative role of *P. vivax* in some of these severe manifestations remains to be proven and many are sufficiently rare that they are unlikely to represent significant public health problems. Anaemia, on the other hand, is a common and frequently severe consequence of vivax infection [14-16,25-27]. This review explores the epidemiology, pathophysiological mechanisms, relationship to transmission dynamics and consequences of anaemia caused by vivax malaria (highlighting similarities and differences as compared with *P. falciparum*). The impact of anti-malarial treatment on haematological recovery is also described.

### Epidemiology

Endemic *P. vivax* is transmitted throughout the tropics in much the same geographical pattern as *P. falciparum*[1,8]. The major exception is West Africa where *P. vivax* is largely absent. This has been attributed to selection over many millennia of individuals lacking the Duffy red blood cell surface antigen - an important mediator of *P. vivax* red cell invasion [2,28]. Menard and colleagues’ recent discovery of vivax malaria in Duffy negative Malagasy people suggests that *P. vivax* may have escaped its dependence on the Duffy antigen in certain regions [29].

Where *P. falciparum* and *P. vivax* co-exist, both the incidence of infection and severity of haematological morbidity attributable to *P. vivax* tends to peak at a younger age than for falciparum malaria and in many regions, before the end of the second year of life [16,27,30-32]. This pattern probably reflects greater parasite exposure through frequent relapse and consequently more rapid acquisition of immunity to vivax malaria. In tropical regions, relapses may occur as often as every three weeks [33,34]. In Papua, Indonesia, an area of comparatively high-level *P. vivax* transmission, approximately a quarter of infants hospitalized with vivax malaria have severe anaemia (haemoglobin less than 5 g/dL), whereas in individuals 45 years or older this proportion decreases to less than 5% [16]. Infants with vivax malaria in this region have 2.4-fold greater odds of severe anaemia than those with falciparum malaria [27].

Females are at greater risk of hospitalization with *P. vivax* malaria than males [16] and in one large analysis were more likely to present with anaemia (unpublished data). Both phenomena may be, at least partially, explained by the fact that post-pubertal women have a lower mean haemoglobin concentration than men and therefore have a greater chance of being tipped over the threshold for anaemia following a haematological insult. Pregnant women with *P. vivax* infection have a ~2-fold higher risk of moderate anaemia than uninfected pregnant women [35-37]. Whether the haematological impact of vivax malaria in pregnancy is greater than in non-pregnant women of child-bearing age is unknown.

Several red blood cell and haemoglobin variants have been associated with reduced susceptibility to anaemia caused by falciparum malaria, the best known being sickle cell anaemia. Hypothesized mechanisms for this protection include reduced red blood cell invasion, relative inhibition of intracellular parasite replication, more efficient removal of infected red blood cells by the spleen and more efficient presentation of parasite antigens to the immune system [38-40]. The high erythrocyte counts and relative microcytosis seen in conditions such as alpha-thalassaemia result in a lower proportional reduction in haemoglobin with falciparum malaria [41]. Both alpha- and beta-thalassaemia have been associated with an increased risk of *P. vivax* parasitaemia in cross sectional studies but their effect on vivax anaemia is unknown [42-44]. Preliminary work suggests South-East Asian ovalocytosis may protect against *P. vivax* parasitaemia and vivax-associated anaemia [45,46] whereas the effects of Gerbich blood group are unclear [47]. Glucose-6-phosphate dehydrogenase deficiency is associated with protection against clinical disease and reduced parasite density in *P. vivax* infections [48,49]. The rarity of such polymorphisms in migrant Highland Papuan populations has been hypothesized to contribute to the higher risk of severe anaemia from *P. vivax* in Southern Papua compared with elsewhere in New Guinea [22].

Gastrointestinal helminth infections may cause anaemia through chronic blood loss, but the interaction with malarial anaemia is complex. In Africa, hookworm and *P. falciparum* malaria coinfection has been shown to cause an additive reduction in haemoglobin in children and pregnant mothers when compared with monoinfection with either parasite alone [50]. Helminthiasis may also be a risk factor for *P. falciparum* parasitaemia but evidence is conflicting [51,52]. Few studies have addressed the effect of intestinal helminthiasis on the risk of *P. vivax* infection and vivax-associated anaemia. Boel and colleagues showed a positive association between *Ascaris lumbricoides* infection during pregnancy and risk of vivax malaria [53]. Another small study found that the reduction in haemoglobin associated with *P. vivax* infection in children between five and 14 years of age was attenuated by coinfection with hookworm, *A. lumbricoides* and *Trichuris trichuria*[54].

The haematological effects of chronic blood loss caused by intestinal helminthiasis are exacerbated by nutritional iron deficiency which in turn may interact with the haematological effects of *P. vivax* malaria. Iron deficiency is protective against *P. falciparum* infection whereas iron supplementation increases the risk of falciparum malaria and high parasitaemia infections [55,56]. The evidence for a link between iron supplementation and morbidity associated with vivax malaria is conflicting. One large prospective study from Thailand showed that pregnant women given supplemental iron and folate were at increased risk of *P. vivax* infection compared to those who did not receive supplementation [57]. A randomized controlled trial from Peru showed that iron plus zinc reduced vivax-associated morbidity in children under five years of age, but iron supplementation increased morbidity in those over five years [58]. In Papua New Guinea, 16 weeks of supplemental iron in prepubescent school children provided an overall haematological benefit compared with placebo and had no effect on the risk of morbidity associated with vivax malaria [59]. Large, multi-centre trials including both pre- and post-pubescent participants are required to firmly establish whether an association between iron status and vivax anaemia exists.

### Pathophysiology and mechanisms

**Figure 1:**
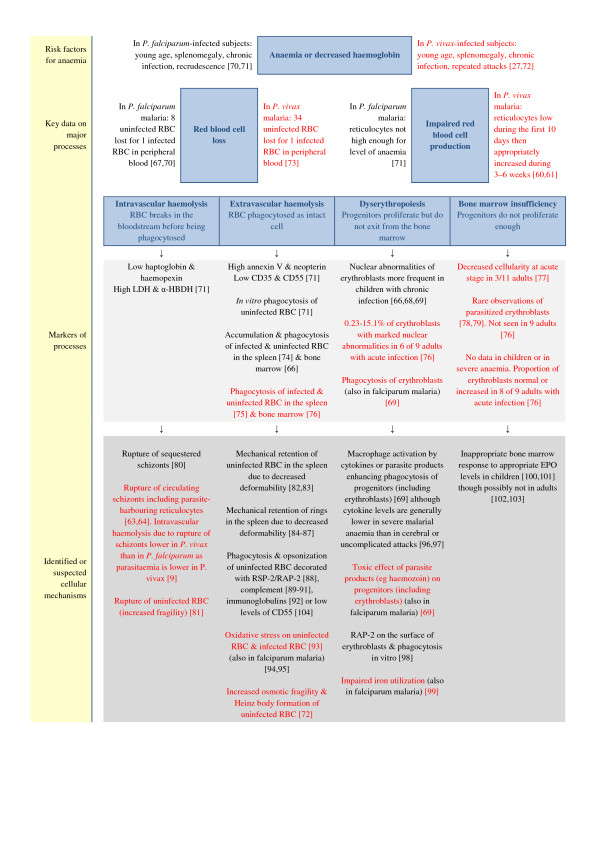
**Comparative pathogenic mechanisms of anaemia associated with*P. vivax*and*P. falciparum*malaria (mechanisms in red font relate to vivax malaria and mechanisms in black relate to falciparum malaria).** Abbreviations: RBC; red blood cell, CD35; complement receptor 1, CD55; decay accelerating factor, LDH; lactate dehydrogenase, α-HBDH; alpha-hydroxybutyrate dehydrogenase, RSP-2; ring surface protein 2, RAP-2; rhoptry-associated protein-2, EPO; erythropoietin.

The primary target of human *Plasmodium* species is the red blood cell. *Plasmodium vivax* has a very strong predilection for red blood cells that have emerged from the bone marrow within the last 14 days, in particular reticulocytes, whereas *P. falciparum* has only a moderate predilection for young red blood cells and significant ability to infect older cells [60-62]. The natural history of erythrocytes infected by either species is to host the replicating parasite for approximately 48 hours before bursting and releasing daughter merozoites. The range of peripheral parasitaemia in *P. vivax* infections is lower than in symptomatic *P. falciparum* malaria and parasitaemia >2% is rare [9]. Despite this, mathematical models suggest that premature death of infected reticulocytes due to *P. vivax* infection should be sufficient to lead to extreme anaemia over a period of several months by choking the supply of mature red blood cells [63-65]. Direct evidence from two malariatherapy patients studied in detail shows that severe anaemia may develop much more rapidly than this and that the proportion of infected reticulocytes after two to three weeks of vivax malaria can be less than 10% [60]. These observations suggest that other mechanisms of anaemia are likely to be important. In *P. falciparum* malaria, these include increased removal of infected, and to a greater extent, uninfected red blood cells from circulation, compounded, in subacute and chronic forms, by impaired erythropoiesis [66-70]. The same general processes appear to be important in vivax malaria but many of the cellular mechanisms differ (see Figure 1[9,27,60,61,63,64,66-104]).

### Removal of red blood cells from circulation

Although parasitaemia is typically lower in vivax compared with falciparum infections, the absolute number of red blood cells removed from circulation, and hence the degree of anaemia resulting from infection by the two species, is often similar [16,26,105]. This is because in *P. vivax* malaria, approximately 34 non-infected cells are cleared for every one infected cell [73] whereas in *P. falciparum* malaria, this ratio is closer to 8 to 1 [67,70]. These figures are derived from observations in non-immune adults treated for neurosyphilis [67,73] and Thai patients over 5 kg in weight [70]. There are no data on the proportional removal of uninfected red blood cells in infants, the age group bearing the greatest burden of anaemia due to vivax malaria. Although removal of uninfected red blood cells is an important component of vivax-associated anaemia, the mechanisms of removal are not fully understood.

As in falciparum malaria, vivax-infected erythrocytes adhere to uninfected red blood cells (rosetting) [106,107] but unlike falciparum-infected cells, they have limited propensity to adhere to endothelial cells and, therefore, sequestration in the deep microvasculature is not a major factor in the pathogenesis of vivax malaria [108,109]. Erythrocytes parasitized by *P. falciparum* become less deformable than uninfected red cells and have reduced capacity to pass through narrow inter-endothelial slits in the wall of splenic sinuses (mean dimensions 1.89 × 0.65 μm) [85-87,110]. Vivax-infected cells become more deformable as the parasite matures and are thought to retain the ability to squeeze through splenic slits [81,110,111]. In falciparum malaria, red blood cell sequestration reduces the proportion of parasitized red blood cells that traverse the spleen. Increased deformability of infected red blood cells in vivax malaria may limit the proportion of red cells that are removed during passage through the splenic microcirculation. Thus, it appears that *P. falciparum* and *P. vivax* have evolved two different means of escaping splenic filtration. In both vivax and falciparum malaria, parasitized, and possibly non-parasitized, red cells are hypothesized to be more fragile than red cells in non-infected individuals and more prone to damage from shear stresses [72,81,110,112]. This process is potentially a more important cause of red cell loss in falciparum malaria since in this disease, major sequestration in the microvasculature impedes the passage of circulating erythrocytes and erythrocyte rosettes [82].

In addition to these mechanical processes, activation of the innate, cell-mediated and humoral immune systems in response to the presence of *P. vivax* antigens enhances the detection and removal of infected and abnormal uninfected red blood cells [69,75,113]. The non-specific immune response for a given parasitaemia is greater for *P. vivax* than *P. falciparum* and may partially explain the greater proportional removal of non-parasitized cells and lower fever threshold in vivax malaria [114-116]. This is a relatively weak speculation however since in severe falciparum malarial anaemia, cytokine levels are generally lower than in cerebral or uncomplicated attacks [96,97] and cytokine concentrations have not been found to correlate with the degree of anaemia in *P. vivax* infections [105]. Macrophage hyperplasia and increased phagocytic activity in both falciparum and vivax malaria results in a highly oxidative environment and may contribute to the shortened lifespan of non-infected erythrocytes [75,117-121]. To compound the problem, reduced glutathione, which is necessary for protecting red cells against damaging oxygen species, is reported to be depleted in vivax malaria [93,122]. Infection with *P. falciparum* causes altered expression of complement components and deposition of parasite proteins on infected and uninfected red blood cells [88,92] (the latter sometimes associated with presence of specific immunoglobulins); facilitating opsonization and complement-mediated phagocytosis [89,91,123]. It is unknown whether these processes also occur in vivax anaemia.

Whatever the mechanisms leading to red blood cell alteration, the spleen is the most important site for the filtration, retention and phagocytosis of non-sequestered erythrocytes parasitised or altered by *P. falciparum*[87,124-127]. Splenic activity limits parasite density thereby reducing the risk of severe malaria. However, the more stringent the splenic clearance, the greater the likelihood of severe anaemia [124,125,128]. This may explain why concomitant severe malarial anaemia with spleen enlargement and cerebral malaria is relatively unusual with cerebral manifestations being more common in acute, fulminant infections and anaemia being more likely in chronic infections [27,76,128,129]. The role of the spleen in vivax malaria is poorly understood though splenic enlargement in this infection appears to be similar to falciparum malaria [130,131]. Indeed vivax malaria carries a very low but well-known risk of splenic rupture; considered greater than for falciparum malaria [132,133]. In 1974, Littman described a single patient with hereditary spherocytosis who developed severe anaemia secondary to vivax malaria. A relapse five months later after a splenectomy did not cause anaemia suggesting that the spleen was the primary site of red blood cell removal (though the effect of strain specific immunity could not be excluded) [134]. A study from Papua showed that plasma haemoglobin concentrations in adults with uncomplicated vivax malaria were not increased compared to controls and were significantly lower than in falciparum malaria (unpublished data). This suggests that in adults with vivax malaria, the degree of intravascular haemolysis may be less than in falciparum malaria and that a greater proportion of uninfected red blood cells undergo extravascular removal.

Increased removal and destruction of both infected and uninfected red cells in vivax malaria is most prominent during the early stages of infection however enhanced removal of uninfected cells persists for five weeks or more after effective treatment of blood-stage infection [135,136]. In chronic, asymptomatic vivax parasitaemia, common in vivax-endemic areas, removal of both infected and uninfected red cells is likely to persist for the duration of infection.

### Impaired production of red blood cells

Patients with vivax or falciparum malaria have bone marrow abnormalities reflecting impaired erythropoiesis. In the earliest stages of both infections, the typical marrow finding is of decreased cellularity [69,77]. In those with more chronic infections, marrow cellularity tends to be normal or increased but there is ineffective erythropoiesis [66,68,76], as indicated by impaired iron utilization [69,99], presence of morphologically abnormal erythroblasts as a result of cellular injury [76], and phagocytosis of erythroblasts by marrow macrophages [69,76].

The exact mechanisms and functional importance of impaired erythropoiesis in vivax malaria are unclear. Using electron microscopy, Ru and colleagues have shown parasitization and subsequent degradation of erythroblasts in two patients with uncomplicated vivax malaria [78]. Yoeli demonstrated morphologically normal, but non-pigmented, intracellular *P. vivax* parasites in a sternal tap specimen but not in peripheral blood smears in a single patient with vivax malaria [79]. Wickramasinghe did not find any *P. vivax* parasites in the marrows of nine Thai adults with uncomplicated *P. vivax* infections [76]. Because of the absence of any bone marrow data in children, or at any age with severe vivax-associated anaemia, the importance of *P. vivax* parasitization of erythroblasts in severe vivax anaemia is not known. Hypoxia of the bone marrow resulting from obstruction of marrow sinusoids by parasitized red blood cells and inadequate erythropoietin production or response have been hypothesized to contribute to impaired erythropoiesis in *P. falciparum* infections [76,102,103,137-139]. In vivax malaria, hypoxia of the bone marrow is unlikely to be significant as there is minimal schizont sequestration. Erythropoietin metabolism is yet to be studied in this disease.

Wickramasinghe and colleagues proposed that *P. vivax* has a directly toxic effect on erythroblasts or their precursors [76]. Alternatively *P. vivax* may exert its effect on bone marrow macrophages leading to increased phagocytic activity and/or release of locally cytotoxic molecules damaging surrounding haematopoietic cells [76]. Whatever the cause, some degree of impaired erythropoiesis has been shown to persist for at least two weeks after treatment of vivax malaria and therefore the effects of these putative factors must be long-lasting [76].

### Effects of transmission intensity, relapse patterns, and strain diversity

**Figure 2:**
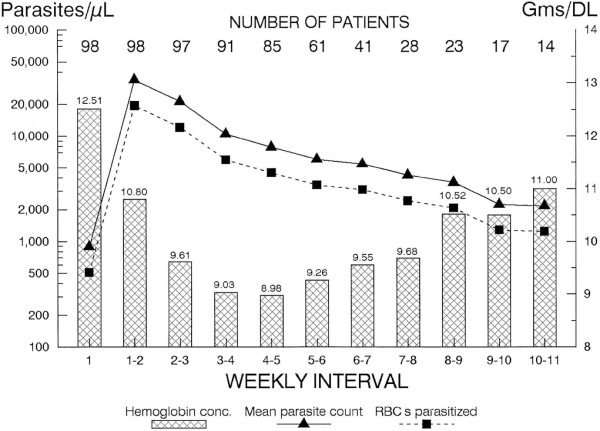
**Mean haemoglobin concentration in relation to parasitaemia in patients with syphilis treated with induced*P. vivax*infections (98 with the St Elizabeth strain, 11 with the Chesson strain and 2 with the Korean strain).** Reproduced with permission from Collins WE, *et al*. [73].

Since a significant proportion of the anaemia of vivax malaria, at least in the acute phase, can be explained by removal of uninfected red cells in response to immune system activation, the magnitude of the immune response (of which intensity of symptoms can be taken as a proxy) is likely to be an important determinant of haematological impairment. Untreated primary sporozoite-induced infection in non-immune adult patients with neurosyphilis typically results in paroxysmal fever lasting 3–8 weeks followed by an extended period of increasing clinical ‘tolerance’ to persistent parasitaemia [140]. Anti-parasite immunity that suppresses parasitaemia to subpatent levels takes significantly longer to develop, in many cases more than 200 days [141]. Collins and colleagues have reviewed the natural history of haemoglobin changes associated with untreated *Plasmodium vivax* infection in adult neurosyphilis patients [73]. There was an exponential decay in mean haemoglobin concentrations during the first 4–5 weeks followed by a gradual climb in concentrations coinciding with development of parasite tolerance (see Figure 2). With persistent infection, mean haemoglobin levels had still not returned to normal by week 11 though the trajectory of the changes suggests that they might eventually have done so [73].

Repeat infection with the homologous *P. vivax* strain, whether due to reinfection, recrudescence or relapse, during the period of parasite tolerance may result in a transient rise in parasitaemia but rarely recurrent or exacerbated symptoms [142-144]. Homologous re-exposure after an extended period of parasite exposure (approximately 6 months for the St Elizabeth strain [141]) from untreated or inadequately treated primary infection, relapse or reinfection results in neither patent parasitaemia nor clinical symptoms [142,143,145-148]. Since homologous re-exposure in tolerant or immune individuals causes little non-specific immune system activation, the haematological effects of this are likely to be minimal.

Heterologous immunity however, is weak and exposure to a new strain following primary infection will usually result in clinical malaria with non-specific immune system activation, recurrent symptoms and presumably a repeat haematological insult [144,149]. If this occurs before an individual’s haemoglobin concentration has returned to normal following primary infection, the haematological effects are likely to be additive though direct evidence supporting this postulate is limited [33,72,150]. The likelihood of repeat heterologous infection, and the probability that it occurs before complete haematological recovery, is a function of, among other things, the relapse pattern of local *P. vivax* strains and the entomological inoculation rate, both of which are also likely to affect the rate of genetic recombination and hence parasite population diversity [151].

In endemic regions, a high proportion of *P. vivax* relapses are known to be caused by heterologous strains (with regard to the previous vivax infection) [152,153]. This is likely to be particularly common in areas with relatively intense *P. vivax* transmission and/or extensive parasite strain diversity. In such regions, there will also be a greater chance of simultaneous inoculation of two different strains of *P. vivax* which has been shown to cause deficient homologous immunity and therefore presumably greater susceptibility to the haematological effects of future homologous infection [154].

Different *P. vivax* strains have markedly different relapse patterns and in some instances may not even cause a primary infection [34,141,155]. In tropical regions, relapses tend to occur regularly every three to four weeks and rarely persist for more than four years from the time of initial inoculation [33]. In temperate climes, the period between relapses may be significantly longer and more variable with some strains showing a bimodal frequency pattern [33]. Tropical strains with short relapse intervals will be more likely to cause repeat blood stage infection before complete haematological recovery has occurred and may therefore be expected to have the greatest haematological impact.

The virulence of an individual parasite strain may also modulate its haematological effects. There is some evidence that the Chesson strain causes fever at lower parasitaemia than other strains and therefore that it may be more immunogenic [156]. In 1947, Whorton and colleagues described the anaemia associated with this strain as “striking” stating that “after the second week of malaria [in malariatherapy patients], it was difficult to maintain patients’ erythrocyte count above 2 million per cmm [corresponding to a haemoglobin concentration of approximately 6 g/dL] in spite of frequent erythrocyte transfusions” [156].

Given the aforementioned evidence, one might predict that the severest haematological impact of vivax malaria would be seen in tropical regions with high entomological inoculation rates and virulent circulating strains. These features are perhaps best exemplified by *P. vivax* on the island of New Guinea where the frequently relapsing Chesson strain is endemic and local populations experience particularly severe haematological impairment from vivax malaria [14-16,27]. In these regions, broad anti-disease immunity to all circulating parasite strains is also likely to develop more rapidly and therefore the haematological burden of vivax malaria will be most heavily skewed to very young children [30].

Most populations at risk of *P. vivax* infection are also exposed to *P. falciparum* and co-infection (not necessarily patent) with these two species is common [150,157-159]. In Thailand, mixed infection with *P. falciparum* and *P. vivax* has been shown to attenuate the risk of severe anaemia associated with falciparum malaria – possibly due to some degree of cross-species immunity [70,160-162]. Recent work has shown that in Papua New Guinea and Papua, Indonesia, mixed infection causes more severe haematological impairment than infection with either species alone [14,16,27]. The explanation for these opposing findings probably lies in the different transmission dynamics in these regions. In Thailand, severe falciparum malaria is usually the result of a single, fulminant infection in a non-immune individual and therefore earlier induction of suppressive non-specific immunity provided by coincident *P. vivax* infection, however minor, is likely to have a protective effect. In New Guinea, where transmission is more intense, severe anaemia in those older than one year is more likely to be the result of repeated or continuous infections due to either species. In this situation, the haematological effects of infection with both species are more likely to be additive and any immunomodulatory effects relatively minor.

### Effects of anti-malarial treatment

Early treatment of malaria can truncate the impending reduction in haemoglobin and accelerate haematological recovery [70,163,164]. Despite the overall benefits of treatment, haemoglobin typically falls slightly following initiation of an anti-malarial drug reaching a nadir between days three to 7 following treatment [70,165]. Data are sparse but there is some evidence that this initial fall may be less pronounced following treatment of vivax malaria with chloroquine (which gives faster clinical and parasitological responses against sensitive strains) compared with either sulfadoxine + pyrimethamine or chlorproguanil + dapsone [165]. The artemisinin derivatives cause an extremely rapid reduction in *P. vivax* parasite biomass. They also temporarily reduce red blood cell production [166-168]. In falciparum malaria, evidence suggests that the haematological benefit of the greater efficacy of the artemisinin drugs negates or outweighs the detrimental effects of this bone marrow suppression [169,170]. The only published comparative assessment of the acute haematological effects of treatment with an artemisinin derivative in patients with vivax malaria showed that artesunate + pyronaridine was associated with a greater mean reduction in haemoglobin at days 3 and 7 when compared with chloroquine alone, although no tests of statistical significance were given [167].

Complete removal of blood stage parasites following blood schizontocidal treatment allows faster haematological recovery (pre-infection haemoglobin concentrations are generally achieved in approximately 4–5 weeks following effective treatment [70,163,171,172]) but reduces total parasite exposure and hence limits the development of homologous immunity [146]. Treated individuals are therefore more likely to develop clinical malaria with significant haematological impairment following repeat homologous infection [146,148,173]. Highly efficacious blood schizontocidal regimens containing slowly eliminated drugs (such as chloroquine, piperaquine or mefloquine) minimize the risk of recrudescence and also provide extended post-treatment prophylaxis against recurrent infection, allowing more time for full haematological recovery [5,150,163,174,175]. The haematological benefit of the long elimination half-life is likely to be greatest in equatorial regions where *P. vivax* strains relapse as often as every three weeks.

*Plasmodium vivax* has developed high-grade resistance to chloroquine in parts of Oceania, Asia, Africa and Latin America [4,5] and sulfadoxine + pyrimethamine in parts of South-East Asia [176]. Clinically, drug resistance is manifest by delayed parasite clearance times, an increased likelihood of incomplete parasite clearance and subsequent recrudescence as well as a shorter period of post-treatment prophylaxis against early recurrence [5]. These factors are likely to result in a greater haematological insult associated with the initial infection (as demonstrated in *P. falciparum* malaria [70,164,177]) but may facilitate earlier development of anti-disease immunity.

Primaquine is a hypnozoitocidal drug that, if administered correctly, can prevent *P. vivax* relapses and thus reduce the total haematological impact of a given infection. Unfortunately this medication has the potential to cause lysis of old red blood cells in all patients, but particularly those with glucose-6-phosphate dehydrogenase deficiency [178,179]. G6PD deficiency is the most common heritable enzymopathy in the world, with a prevalence ranging from 7.5% in Africa as a whole to 2.9% in the Pacific [180]. Although G6PD deficiency increases the susceptibility of erythrocytes to oxidative damage this alone is not sufficient to account for primaquine-induced haemolysis [181,182]. Generally, the more severe the enzyme deficiency, the greater the severity of haemolysis [178,179]. Individuals who have less than 10% of normal enzyme activity are at risk of life-threatening haemolysis after as little as one dose of primaquine [183] whereas those with milder variants may have negligible effects [178]. Weekly, as opposed to daily, dosing schedules mitigate primaquine-induced haemolysis [184] whilst retaining efficacy [185] though adherence to such regimens is likely to be poor unless therapy is supervised. In mildly deficient individuals, continuous daily primaquine dosing causes acute but self-limited haemolysis for approximately 10 days followed by reactive reticulocytosis and haematological recovery as the population of old, susceptible, red blood cells is replaced by young erythrocytes [184]. Since this is not an immunological phenomenon, repeat challenge with primaquine after a period of time in such patients causes equally severe haemolysis [179]. In severely deficient patients, haemolysis is progressive and can have a fatal outcome unless primaquine therapy is stopped and blood transfusion given [186,187].

### Consequences of vivax anaemia

The impact of *Plasmodium vivax* infection on haemoglobin concentration varies from negligible to dramatic [10,14-16,188,189]. The clinical consequences of the reduction in haemoglobin depend on the haemoglobin concentration prior to infection. For example, an absolute reduction of 2 g/dL would be more likely to have dramatic consequences if the initial haemoglobin was 6 g/dL than if it was 12 g/dL.

In Papua New Guinea, 1.6% of children under 5 years of age presenting to rural health clinics for treatment of vivax malaria were severely anaemic (haemoglobin <5 g/dL) [14]. Across the border in Indonesian Papua, 22% of patients of all ages who were admitted to hospital with vivax malaria fulfilled criteria for severe anaemia [16]. In the D’Entrecasteaux Islands off Papua New Guinea, a cross-sectional survey of children between 0 and 6 years of age showed that the mean haemoglobin in those with *P. vivax* parasitaemia was 8.7 g/dL, 0.3 g/dL lower than the equivalent value for those infected with *P. falciparum*[190]. In contrast, on the Thai-Myanmar border, less than 0.2% of patients presenting for treatment of vivax malaria were severely anaemic [189].

Although the spectrum of anaemia seen with vivax infection is reasonably well documented, the clinical, developmental, and socioeconomic consequences are largely unknown. Severe anaemia in isolation is associated with a ~2-fold increased risk of death in African children with falciparum malaria and has an even higher mortality when combined with other manifestations of severe disease such as cerebral malaria or respiratory distress [129]. Severe anaemia of any cause in hospitalized children under five years has been associated with a case fatality of between 2% and 29.3% and moderate or severe anaemia has been associated with a maternal case fatality of between <1% and >50% in hospital-based studies [191,192].

Population-based estimates of mortality in severely anaemic individuals with vivax malaria have not been established but recent studies from Latin America, New Guinea and the Indian subcontinent have identified deaths in patients with severe vivax anaemia [10,15-17]. The authors did not establish the extent to which anaemia contributed to those deaths.

Anaemia caused by vivax malaria is associated with requirement for blood transfusion [188,189]. Screening of blood products for pathogens is well known to be incomplete in many low and middle income countries and therefore has a significant attendant risk of pathogen transmission [193]. For example, in Sub-Saharan Africa, estimates for the risk of transfusion-associated infection with HIV, hepatitis B and hepatitis C are 1, 4.3 and 2.5 infections per 1,000 units of blood respectively [194].

Pregnant women with haemoglobin concentrations under 8 g/dL in Papua New Guinea were at 2.4-fold higher risk of delivering a low birth weight baby than non-anaemic mothers [195]. In this study, primigravidae with anaemia and parasitaemia at the time of delivery had the greatest risk of low birth weight [195]. Although vivax malaria is endemic in Papua New Guinea, attribution of these effects specifically to this species is not possible [195]. In Papua, Indonesia, *P. vivax* parasitaemia at delivery is associated independently with an increased risk of moderate anaemia and a mean reduction in birth weight of 108 g [35].

Although evidence is lacking it seems plausible that severe vivax anaemia may reduce resilience to other infectious and non-infectious diseases and therefore may be associated with indirect mortality. In 1938, Swellengrebel and de Buck reported that 62 (7.7%) of a series of 807 patients with syphilis who were treated with induced *P. vivax* infections subsequently died; those with other comorbidities were at particularly great risk [196].

Chronic or repeated episodes of malarial anaemia due to any *Plasmodium* species have been associated with adverse effects on physical and cognitive development as well as school attendance; all of which may be exacerbated by concomitant malnutrition [197-202]. Again, whether these outcomes are generalizable to vivax malaria, and more specifically the haematological effects of this species, is unknown.

## Conclusions

Haematological morbidity associated with *P. vivax* infection is greatest in young children, especially in tropical countries such as Papua New Guinea and Eastern Indonesia where transmission is intense and local parasite strains relapse very frequently. In these regions, vivax malaria is commonly associated with severe anaemia both in the health care and community setting. The haematological effects of vivax malaria are likely to have complex interactions with gastrointestinal helminth infection, haemoglobin and red blood cell abnormalities and malnutrition.

**Figure 3:**
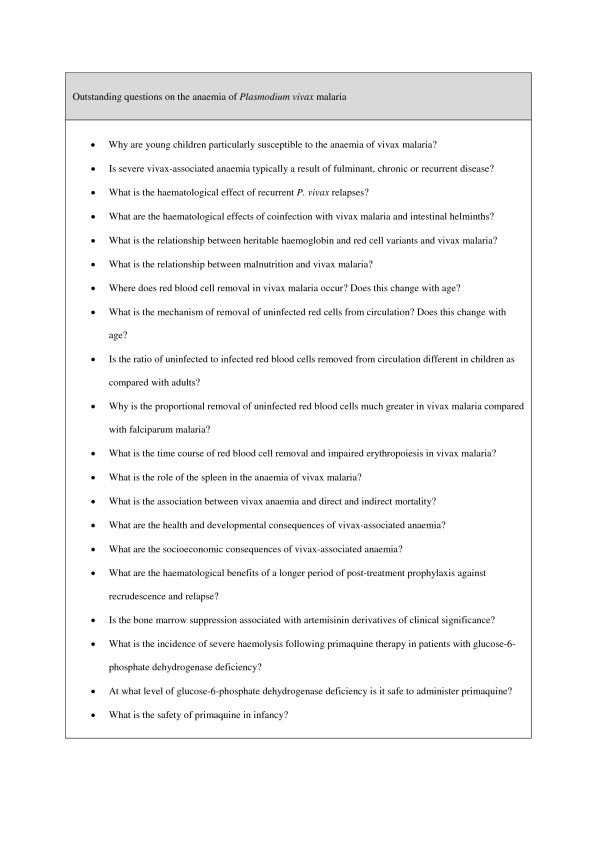
**Outstanding questions on the anaemia of*Plasmodium vivax*malaria.**

Removal of uninfected red blood cells is a particularly important mechanism of anaemia in acute vivax malaria. *Plasmodium vivax-*infected red blood cells are minimally adherent and are more deformable than *P. falciparum-*infected erythrocytes resulting in relatively little red blood cell sequestration in the microvasculature and marrow sinuses and passage of a greater proportion of red cells through the spleen and other reticuloendothelial organs. The role of the spleen in the pathogenesis of vivax anaemia, particularly the removal of uninfected red blood cells, is an important area for future research (Figure 3).

As the global control and elimination of malaria progresses, *P. vivax* is set to become the dominant *Plasmodium* species [4,203], yet the health, developmental and socioeconomic consequences of vivax malaria and vivax–associated anaemia have received very little attention. Severe vivax anaemia may cause significant morbidity and indirect mortality via association with impaired resilience to infectious and non-infectious comorbidities, obstetric complications and requirement for blood transfusion (with attendant risk of blood-borne pathogen transmission). Early treatment with an efficacious blood schizontocide can reduce the initial fall in haemoglobin associated with vivax infection and thus help to prevent adverse outcomes associated with severe anaemia. Reliable prevention of recurrent haematological insults caused by relapses will require hypnozoitocidal therapy. Primaquine is the only licensed hypnozoitocidal agent available and can exacerbate haemolysis in individuals with G6PD deficiency. Policymakers need to weigh the potential benefits of this drug against the risks based on the local prevalence of this enzymopathy as well as the availability of G6PD testing. Vivax-associated anaemia is an important public health concern that underscores the importance of reducing global transmission of *P. vivax*.

## Competing interests

All authors declare they have no competing interests.

## Authors’ contributions

NMD, PAB, NMA and RNP searched the relevant literature. NMD wrote the first draft of the manuscript. All authors appraised and revised the manuscript. All authors gave final approval for submission of the manuscript.
